# Development of Cold Plasma Technologies for Surface Decontamination of Seed Fungal Pathogens: Present Status and Perspectives

**DOI:** 10.3390/jof7080650

**Published:** 2021-08-11

**Authors:** Jure Mravlje, Marjana Regvar, Katarina Vogel-Mikuš

**Affiliations:** 1 Biotechnical Faculty, University of Ljubljana, 1000 Ljubljana, Slovenia; marjana.regvar@bf.uni-lj.si (M.R.); katarina.vogelmikus@bf.uni-lj.si (K.V.-M.); 2 Jožef Stefan Institute, 1000 Ljubljana, Slovenia

**Keywords:** cold plasma, seeds, grains, microorganisms, fungi, agriculture, plant production, food security

## Abstract

In view of the ever-growing human population and global environmental crisis, new technologies are emerging in all fields of our life. In the last two decades, the development of cold plasma (CP) technology has offered a promising and environmentally friendly solution for addressing global food security problems. Besides many positive effects, such as promoting seed germination, plant growth, and development, CP can also serve as a surface sterilizing agent. It can be considered a method for decontamination of microorganisms on the seed surface alternative to the traditional use of fungicides. This review covers basics of CP technology and its application in seed decontamination. As this is a relatively young field of research, the data are scarce and hard to compare due to various plasma setups and parameters. On the other hand, the rapidly growing research field offers opportunities for novel findings and applications.

## 1. Introduction

According to The Food and Agriculture Organization of the United Nations (FAO) predictions, the human population will reach almost 10 billion by 2050, making global food security one of the greatest challenges of our time. It is estimated that the production of food crops must increase up to 70% to meet the needs of a rapidly growing population [1]. The expansion of food production and economic growth is closely linked to environmental issues such as deforestation, loss of biodiversity, depleted groundwater resources, and greenhouse gas emissions, which contribute to climate change [2]. Moreover, the amount of land available for cultivation is limited and declining due to drought, desertification, salinization, erosion, etc., so future research activities need to focus on improving and increasing the efficiency of food production and processing.

One of the main concerns in most reported foodborne illness outbreaks involves contaminated cereal grain [3]. Cereal contamination can occur at many stages: postharvest transport, conditioning, sorting, storage, and packaging [4]. Fungal contamination is a major safety problem in stored cereals [5], as it not only spoils the grain but also reduces its cooking or baking quality, lowers its nutritional value, and alters its overall appearance by causing unwanted odours and colours [6]. Furthermore, some fungi also produce toxic substances, such as mycotoxins, with aflatoxins (produced by the genus *Aspergillus*) being the most toxic [7,8]. For this reason, it is essential to protect crops, seeds, and their food products from rot and/or pests [5].

Fungi can infect seeds either in the field or during storage if conditions are favourable. The first group can be classified as preharvest fungi, with major genera *Alternaria, Fusarium, Cladosporium, Rhizopus* etc., and the second as postharvest fungi, with genera *Aspergillus* and *Penicillium* being predominant [9]. Traditionally, seeds are mainly treated by fungicides, the most effective and widely used method to prevent and suppress fungal growth [10]. Conventional treatment of seeds also includes different types of physical treatment with the most common thermal treatment that inactivates the pathogen and leaves the seed tissue viable [11]. In the light of reducing the usage of pesticides, which are harmful to humans and the environment, and with the growing demand for “pesticide-free food”, in the past century treatments with biopesticides have become popular [10,12]. However, even these methods are not always effective and have their shortcomings, as some CP systems are still not cost-effective for mass production. Therefore, additional research in this field is necessary to achieve environmentally friendly and economically feasible alternatives for pest management, which are needed both in pre- and postharvest production. The emerging use of CP technology offers promising solutions for the inactivation and elimination of fungi and their toxins. As a form of “non-thermal” processing technology, where the treated objects are not exposed to excessive heat treatment, exposure to cold plasma minimizes the impact on the quality and nutritional value of the products, while maintaining high quality and additive-free products [5]. Over the last 20 years of research in the field of plasma agriculture, numerous studies have shown that treatment with CP can have a positive effect on seeds by improving the germination capacity, seed vigour, growth, and overall performance of plants grown from plasma pre-treated seeds [13,14,15,16,17,18,19,20,21,22]. It has also been found that plasma treatment causes microbial inactivation, thereby suppressing the growth of bacterial and fungal pathogens responsible for various plant diseases [4,5,6,23,24,25,26]. In this perspective, CP technology could be a successful tool for improving seed quality by promoting germination and surface decontamination for seeds (plant embryonic structure for reproduction; in agriculture used for sowing and plant breeding) and grains (plant seeds used as a food source for human or animal consumption). The importance of plasma technology has also increased in recent years in the food industry, as it appears to offer many new applications, i.e., opportunities for new ‘in-package’ food technologies [23,25,27].

This review covers the basics of CP technology, emphasizing its use in the field of agriculture. The main focus is on fungal seed decontamination, covering most studies performed up to date, with concluding remarks focused on the requirements for further research in the field.

## 2. The Basics of Plasma Technology

Plasma refers to the fourth state of matter, an ionized gas with unique properties. It is the most common state of matter in space, since it accounts for more than 99% of the entire universe [28]. Plasma is defined as a “quasi-neutral” medium with a neutral net charge [25], but it is electrically conductive (as it contains free charge carriers) and has many properties that distinguish it from neutral gasses and liquids [28,29]. It consists of electrons, atoms, ions, radical species, and molecules, such as reactive oxygen species (ROS) and reactive nitrogen species (RNS), in a fundamental or excited state, together with electromagnetic radiation (UV photons and visible light). The chemically active species have a pronounced antimicrobial effect and can be used for surface sterilization [5,28].

### 2.1. Generation of Plasma

Plasma is created by applying energy to gas, inducing the formation of ions (charge carriers). Ionization is achieved by supplying thermal energy or applying electric current or electromagnetic radiation [30]. Plasmas can be classified according to the type of energy input, defining the plasma in terms of electronic density and temperature [28].

Depending on the conditions of generation, two plasma categories can be defined, based on the relative energy levels of light (electrons, photons) and heavy particles (ions, molecules) in the plasma [26]. The first group is the so-called “local thermodynamic equilibrium” (LTE) or “thermal” plasma, which has a high electron density and an electron temperature almost equal to the temperature of the heavy particles. The second group is “non-local thermodynamic equilibrium” (non-LTE) or “cold” plasma (CP), which has a lower electron density and a lower temperature because the temperature of heavy particles is much lower than the temperature of the electrons [28]. CP is generally referred to as “low-temperature plasma” (LTP) or “non-thermal plasma” (NTP). This can be further divided into quasi-equilibrium plasma (with a temperature around 100–150 °C) and non-equilibrium plasma (with a temperature below 60 °C). The latter is particularly interesting from a biological point of view, because it can be used to treat living matter without causing thermal damage. CP can be generated at atmospheric or reduced pressure and requires less energy compared to LTE. It can be generated by electric discharge with direct (DC) or alternating current (AC) (with frequencies up to 100 kHz), radio frequency (RF) discharge (with frequencies of 100 kHz–100 MHz), or microwave (MW) discharge (with frequencies above 100 MHz) [5,28].

#### 2.1.1. Electrically Induced Plasmas

Electrically-induced plasma is generated by electric breakdown and is the most widely used method of generating plasma in the presence of an electric field [30]. Although gases are electrically neutral and act as insulators that do not conduct electricity, there is always a certain amount of stray charges in neutral gases. These are crucial in electrical discharges, as they can be accelerated to high energy to excite the gas molecules (by applying an electric field) and produce ionizing shocks, and hence, new charged particles. An electric discharge with DC or AC as a power source can be used to generate this type of plasma. The most common types of DC discharges are corona discharge (when the current range is 10^−7^–10^−5^ A), glow discharge (current range 10^−5^–1 A), and arc discharge (when the applied current is bigger than 1 A) [31]. The best known and most commonly used AC discharge, with the advantage of operating at atmospheric pressure, is the dielectric barrier discharge (DBD), in which the two electrodes are covered by an insulating dielectric barrier [32].

#### 2.1.2. Radio Frequency (RF) Induced Plasmas

**Figure 1:**
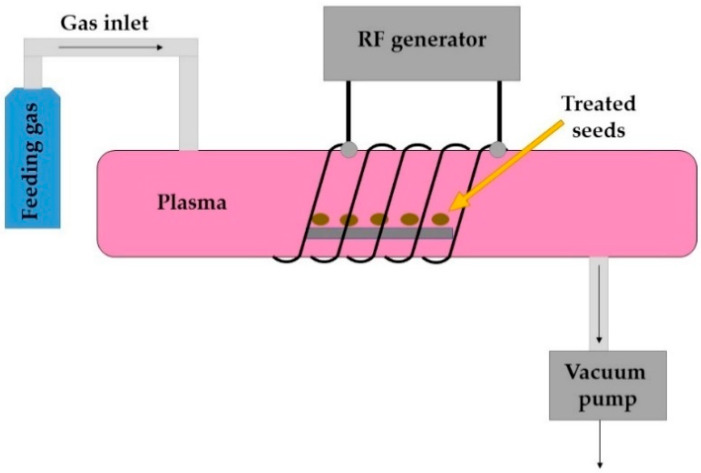
A schematic presentation of a RF-generated low-pressure CP system (adapted by Mravlje et al. 2021 [33]).

During RF discharge, the electrons move back and forth between the electrodes as the field changes direction. This allows a more uniform plasma to be formed. Two types of electrical configuration can be used: capacitively coupled (CC) or inductively coupled (IC). The latter can be carried out as an “electrodeless discharge”, since the electrodes can be placed outside the plasma chamber, thus eliminating contamination of plasma by the electrode materials [31]. An example of RF-generated plasma system is presented in Figure 1.

#### 2.1.3. Microwave (MW) Induced Plasmas

The main difference between MW and other types of discharges is that the electrons absorb the microwave energy collectively (and not individually as in DC or RF) and convert it directly into kinetic energy [31]. Both RF and MW discharges are generated and sustained by high-frequency electromagnetic fields [30].

### 2.2. Plasma Treatment Modes

Biological samples can be exposed to plasma treatment in two different ways: The first is “direct exposure”, where the treated sample is in direct contact with the plasma and thus exposed to all agents generated by the plasma (the UV radiation and all the plasma particles formed in plasma). The second is “indirect exposure”, where the treated sample is placed at a distance or in an adjacent chamber, reducing the amount of heat and preventing many reactive species and charged particles from reaching the sample [34]. Thus, it is not exposed to UV radiation and is affected only by lower concentrations of long-lived plasma species, such as ions, ROS, and RNS [35]. Indirect CP treatment can also be achieved via treatment of seeds with plasma-activated liquids, such as plasma-activated water [36] or plasma-activated sodium chloride [37]. As indirect CP treatment is usually weaker, longer treatment times are needed to obtain similar effects as in direct treatment mode [38].

## 3. Applications of Plasma Technology

CP technology is applied in many different areas of science and technology, including some of our everyday equipment. One of the most well-known applications is in electronics, as used in plasma television (and other display panels) and lighting systems. It is also used in materials science for design and processing of textiles and polymers, and more recently in the synthesis of nanoparticles. Some other areas where cold plasma technology is used also include analytical chemistry (spectroscopic analyses), alternative methods of cleaning (e.g., waste water treatment or soil remediation), and ozone generation. Over the last decade, the use of CP technology in life sciences such as biology, agriculture, food science, and medicine has increased rapidly, creating promising multidisciplinary research areas [5].

### 3.1. Cold Plasma in Agriculture

CP technology seems to be a promising and efficient green technology to improve productivity throughout the food cycle without compromising food quality and safety [39]. Various studies confirmed that CP treatment promotes seed germination, growth, and development in many plant species [22,40,41,42,43,44]. Positive effects of CP treatment on germination rate and/or development and growth have been reported for many economically important agricultural crops such as wheat [14,43,45,46,47,48,49], maize [50,51], soybean [52,53], rice [54], tomato [55,56,57,58], radish [22,59], bean [14,60], barley [20], rapeseed [61], grape [62], mung beans [15], safflower [40], and quinoa [16].

Approaches or techniques that improve seed germination and enhance the growth and overall performance of crops by mitigating soil and environmental stress and/or directly affecting the seed growth process are referred to as “seed priming” techniques [60,63]. The reduction of abiotic stress is often related to the optimization of the seed growth process by altering the availability of water and oxygen in the soil. Seed CP treatment could be considered as one of the seed-priming techniques. It can give different results depending on the plant species studied and the type of gas used for plasma generation [64].

The possible mechanisms of CP treatment on seed germination and growth include: changes in the wetting properties by reducing the contact angle due to surface oxidation [14,65]; stimulation of caryopsis by surface erosion [43]; changes in antioxidant activity such as the production of reduction-type thiol compounds [66]; reduction of abscisic acid content [67]; and changes in other endogenous plant hormones such as auxins, cytokinins, and gibberellins [22,68], all of which are involved in the promotion of plant growth. In short, CP influences seed germination and growth by alternating their physiological and biochemical properties. In addition to the direct plasma treatment, it has recently been shown that the use of plasma-activated water can increase plant growth [47,58,69] and enhance the effect of chemical fertilizers [69].

Apart from beneficial action on seed germination and plant growth and development, one of the outstanding effects of plasma treatment is the sterilization of the seed surface [6,70,71]. Free radicals and reactive oxygen species generated by CP induce bactericidal and fungicidal effects, increasing the overall benefit of the CP technology [39]. The use of CP as a sterilizing agent is an alternative to conventional sterilization methods involving various fungicides, being safe both for the operator and the subjects exposed to the plasma, if the operating conditions and set up is optimal, and environmentally friendly [72]. Moreover, CP treatment can also be used to remove harmful chemicals such as fungicides and other pesticides from the surface of long-stored seeds [64]. However, if seeds are intended for further sowing (preharvest CP treatment), their germination parameters must stay unimpaired.

### 3.2. Cold Plasma for Seed Decontamination

Currently, CP treatments are widely used to activate and achieve desired surface changes of various materials and to decontaminate different kinds of surfaces, especially sensitive biomaterials [18,73]. In the last 20 years, the potential of plasma technology for elimination of biological contaminants has been increasingly investigated, since already the first studies indicated that plasma tends to have very efficient germicidal properties with many advantages over conventional sterilization methods and can therefore be an effective decontamination agent [34,74]. The optimal decontamination time to achieve sterilization depends on several factors: the type or species of microorganisms, the surrounding medium, the power density of the plasma, and the type of gas used to generate the plasma [74]. In this perspective, a new field of research has emerged that requires the collaboration of scientists from different disciplines, such as plasma physicists, biologists, microbiologists, and biochemists.

One of the major issues in agriculture is poorly germinating seeds. Besides physiological traits including primary and secondary seed dormancy [75,76], some of the reasons for low germination rates of various plant seeds are epiphytic and phytopathogenic bacteria and filamentous fungi that contaminate seeds [18]. It has been shown that plasma treatment is an efficient sterilizing agent that can inhibit or kill a wide range of microorganisms on the surface of seeds and stored grains and can thus be used to decontaminate seeds [4,5,6,23,24,25,26,64,70,71,77,78].

**Figure 2:**
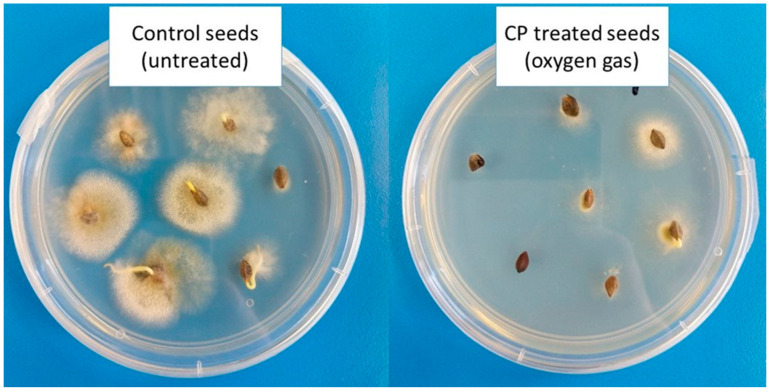
Decontamination of naturally occurring fungi on common buckwheat seeds (*Fagopyrum esculentum* Moench) using radio-frequency-generated LP oxygen CP. Fungal growth after 1 week of cultivation on PDA growth medium is shown [33].

Most studies conducted to date have focused on the microbial inactivation or sterilization efficiency of CP on different bacterial species. However, to assess the efficiency of plasma sterilization, the most resistant microorganisms should be tested [72]. Bacterial endospores and fungi with thick cell walls seem to be the object of interest. It is essential that all possible biological effects that can be caused by plasma are studied in detail and well characterized before CP is accepted as an alternative approach to conventional sterilization methods [26]. It is important to optimize the decontamination process to successfully achieve the elimination of the target microorganisms [5]. We have already mentioned that fungal contamination of stored seeds and cereals is considered an important safety issue, therefore, it is crucial to suppress fungal growth. CP technology offers a promising solution for inhibition of fungal growth (Figure 2), as discussed in the following section.

Most of the commercially used seeds are infected during storage with fungi of various genera such as *Alternaria, Aspergillus, Botrytis, Mucor, Penicillium, Rhizopus, Sclerotinia*, and *Trichoderma* [50,79]. It is known that more than 25 different fungal species infect stored grains and legumes [80], with species of the genera *Aspergillus* and *Penicillium* being responsible for most damage during storage and germination worldwide [6]. Filamentous fungi occur everywhere in nature and pose a potential threat to humans and economically important plant and animal species, as they can contaminate food at various stages of production when temperature and humidity conditions are favourable [81]. The lack of efficient and consistent seed disinfection methods is the main reason for sprout-associated outbreaks with seed-associated microbiota being the primary source of their origin [79].

Fungal species of the genus *Fusarium*, and in particular their mycotoxins, are known to be one of the most important contaminants and as such a major problem in grain and fruit production [5]. Mycotoxins of *Aspergillus flavus* can be very harmful, causing haemorrhage and carcinogenesis [82]. New technologies are needed to reduce or prevent fungal growth without compromising germination and posing safety issues. As CP is considered to have good bactericidal and fungicidal effects on seeds when experimental conditions, variable from species to species, are optimal [50], it could provide an alternative for fungal decontamination of seeds. In Table 1, a review of studies in the field of seed decontamination with both AP and LP CP is shown.

In one of the first studies, the inactivation efficacy of LP CP treatment for two pathogenic fungi (from the genera *Aspergillus* and *Penicililum*) on the surface of various artificially fungal-infected seeds was investigated. It was found that plasma treatment, either with air gas or sulfur hexafluoride (SF6), successfully reduced fungal contamination to levels of less than 1% of the initial fungal load without affecting the germination rate or the quality of the seeds. The best efficacy was achieved with a 15 min exposure to SF6 plasma, with a significant reduction of 3-log * units for both fungal species. (* Log reduction is a measure of how thoroughly a decontamination process reduces the concentration of a contaminant. It is defined as the common logarithm of the ratio of the levels of contamination before and after the process, so an increment of 1 corresponds to a reduction in concentration by a factor of 10). Plasma treatment was confirmed as a fast and functional decontamination method to eliminate aflatoxin-producing fungi from the seed surface [6].

Similar plasma treatment properties (LP CP, air gas or SF6 plasma) were used to investigate the effect of LP CP treatment on the elimination of *Aspergillus parasiticus* from the surface of various nuts. A significant reduction in initial fungal load was observed, as a 5-min air-gas plasma exposure resulted in a reduction of 1-log (and a further 5 min exposure reduced the fungal load for a further 1-log). Again, SF6 plasma proved more effective, resulting in a 5-log reduction of initial fungal load after 5 min plasma exposure. Plasma treatment was found very efficient in aflatoxin reduction. Interestingly, here the air-gas plasma was more effective than SF6, since a 50% reduction of aflatoxin was observed after 20 min of exposure [70].

AP CP was used for decontamination of *Cicer arietinum* seeds [4]. It was shown for the first time that AP CP had a decontamination effect on natural microbiota adhering to the surface of seeds, as a significant reduction of microbial contamination was observed. In addition to the time of AP CP treatment, the effectiveness of microbial inactivation was strongly related to the size and shape of the seeds, with various structures (wrinkles, cracks) on the seed surface forming a barrier and thus having an effect on the decontamination efficacy of the microflora [90,91]. Uniform exposure of the entire seed surface to plasma could be ensured by shaking the seed sample during plasma treatment [4].

In two consecutive studies, the effects of AP fluidized bed (FP) CP system treatment on the effectiveness of surface decontamination of two aflatoxigenic *Aspergillus* species on hazelnuts were studied. The air-gas plasma was more effective in reducing fungal decontamination than nitrogen plasma, with a 5 min exposure to air plasma resulting in a reduction of the initial fungal load in both species by about 4-log (CFU/g). It was also found out that a 2 min plasma exposure resulted in a complete inactivation of the naturally occurring hazelnut microbiota. The AP FB CP system was proposed as a convenient solution for large-scale applications due to its low cost and operational feasibility [83,84].

Effects of AP CP generated by Diffuse Coplanar Surface Barrier Discharge (DCSBD) on wheat germination rate, seedling growth, and inactivation of surface microflora were also studied [18]. DCSBD has been used due to its robustness, safety and applicability in wet and dusty environments, being a good candidate for possible large-scale industrial use. A complete devitalization of the initial count of naturally occurring filamentous fungi occurred during 120 s AP CP treatment of wheat seeds. In the case of artificially infected wheat seeds, representatives of the genus *Fusarium* spp. were most sensitive to AP CP treatment, as complete growth inhibition was observed after a 60 s plasma treatment. Complete inhibition of the epiphytic fungus *Trichothecium roseum* was observed after a 180-s plasma treatment, while species of the genus *Aspergillus* proved to be more resistant. *A. flavus* was completely inhibited after 240 s exposure, while complete devitalization of *A. clavatus* was not observed even at the highest plasma treatment of 300 s.

In a further study, the effects of DCSBD AP CP treatment on maize seeds were investigated [51]. The effectiveness of plasma treatment in decontaminating native microbiota and seeds infected with some phytopathogenic fungal species was evaluated. A higher proportion of contaminants of surface microorganisms belonged to filamentous fungi, with the genera *Aspergillus*, *Fusarium*, and *Penicillium* being the most abundant. The total devitalization of the initial count of naturally occurring filamentous fungi was observed at 180 s plasma exposure, which is comparable to earlier studies on wheat seeds. Of the artificially fungus-infected seeds, *Fusarium culmorum* was again recognized as the most sensitive species, since already a 60 s plasma irradiation led to complete devitalization. On the other hand, complete devitalization of both Aspergillus species was observed after a 300 s plasma exposure (whereas 120-s exposure led to a reduction of 1-log CFU/g of seeds of both species). In addition, no significant changes in the germination rate compared to the control were observed in the 60- and 120-s plasma exposures, whereas the 60-s plasma exposure increased the seedling’s vigour by as much as 23%.

DCSBD AP CP was used to disinfect pine seeds contaminated with the fungus *Fusarium circinatum*, a known quarantine pest of trees, infecting also many species of pine trees. A significant reduction of the fungal pathogen (even complete inactivation at higher exposures) was observed, but unfortunately, the germination rate of pine seeds was also reduced at all plasma exposures, with seeds exposed for more than 60 s not germinating at all [85].

Some studies focused on the effects of CP treatment on naturally occurring fungal microbiota. One of the first studies investigated the fungicidal effects of AP plasma treatment on seeds of some economically important crops and legumes [86]. Here, 10 and 15-min CP treatments were the most effective in reducing fungal infection (by 6–14% of the initial fungal load) and enhancing seed germination. Results were comparable to those obtained with chemical fungicide pre-treatments of seeds.

In a study focusing on the effects of AP CP on native fungal colonies of winter wheat [87], the optimal exposure, where the best reduction of fungal colonies was observed, was 10-s plasma exposure. SBDB AP CP treatment was also used to remove the native microbial contamination in sweet basil (*Ocimum basilicum*) [88]. The plasma treatment significantly reduced fungal contamination, especially during the longest exposure. *Alternaria* was the most common (82%) out of nine different isolated fungal genera naturally occurring on sweet basil seeds. AP CP generated by corona discharge plasma jet (CPDJ) was used for microbial decontamination of rapeseed seeds [61]. All microorganisms tested (including various bacterial species, yeasts, and molds) were reduced in the range of 1.2–2.2 log CFU/g after 3 min of treatment with CDPJ. Plasma treatment up to 2 min also resulted in an improved germination rate and increased seedling growth. In all the above-mentioned research, air was used as a feeding gas for plasma generation.

In a recently published work, the effect of AP DBD CP of argon and argon/oxygen mixture on decontamination of ginsengs seeds was studied [89]. With the mixture, better fungicidal effects were achieved than with pure argon gas. Recently, LP RF-generated oxygen CP was first used to treat naturally contaminated seeds of common and Tartary buckwheat [33]. A significant reduction of seed-borne fungi (to less than 50% of control) and changes in their diversity were achieved after the longest (120 s) CP treatment. However, longer CP treatment also negatively affected germination, so the treatment could be applicable in grain production (postharvest), but not for sowing.

### 3.3. Molecular Mechanisms behind Microbial Decontamination

Plasma type and its properties are crucial for efficient seed sterilization [78]. In contrast to conventional sterilization methods, CP sterilization results in non-linear shapes of survival curves, indicating that CP sterilization is a very complex process in which many factors influence the kinetics of microbial inactivation [92]. CP sterilization can be carried out both in the glow (direct exposure) and afterglow (indirect exposure) regions of the plasma, the former leading to shorter sterilization times and the latter being safer, easier, and cheaper to handle [72]. CP is a highly effective method for inactivation of microorganisms, since antimicrobial agents generated by CP attack many cellular targets so that the development of resistance mechanisms is very unlikely or only possible to a limited extent. The influence of heat during CP treatment is minimal, if not negligible, as it is assumed that the temperature of the sample remains close to the ambient temperature or in any case below the value that could cause thermal damage to the cells, as ions and neutral molecules in the plasma remain relatively cold [34,93].

There is a general agreement that plasma-generated reactive agents trigger a complex sequence of different biological interactions in microorganisms, with reactive species playing an important role in antimicrobial efficacy [5]. However, different studies have drawn various conclusions about the main antimicrobial agents present in plasma that cause inactivation of microorganisms, with the operating pressure at which CP is generated being recognized as one of the main factors determining the predominant antimicrobial mechanisms. Most of the research on molecular mechanisms governing sterilization was performed on bacteria, but it is agreed that similar processes take place in fungi.

#### 3.3.1. Effects of Reduced or Low-Pressure Plasmas (LPP)

Moisan et al., 2001 [72] were among the first to conduct detailed studies on the effects of LP plasma on bacteria. They found that the presence of certain reactive species and UV emitters in the gas is strongly dependent on the pressure of the operating system. They concluded that UV photons play a key role in microbial inactivation of plasma generated at medium and low pressure (≤10 torr). In contrast, during operation at higher pressures up to atmospheric pressure, a large proportion of the UV photons can be reabsorbed by the ambient gas in the plasma, so that they cannot penetrate the samples and act as sterilizing agents. The authors proposed a mechanism of plasma sterilization in an oxygen-containing operating system with reduced pressure, consisting of three basic processes:

1. Inactivation of the genetic material by UV radiation: direct destruction of the genetic material of microorganisms by UV radiation (a statistical process requiring a sufficient number of DNA strand lesions).
2. Intrinsic photodesorption (photon-induced desorption): In this process, UV photons break chemical bonds in microorganisms, resulting in atom-by-atom erosion of the microorganisms and the formation of intrinsic small volatile molecules (such as CO and CHx) as by-products.
3. Etching: a result of the adsorption of reactive species from the plasma onto the surface of microorganisms, resulting in the formation of small volatile compounds (CO_2_, H_2_O). This mechanism can be enhanced by UV photons (“UV-induced etching”), acting synergistically with reactive species and even accelerating the elimination rate of the microorganisms. In the absence of reactive species (e.g., O-atoms), only the initial erosion process (intrinsic photodesorption) can take place, so that a much longer time is required for sterilization (Adapted from [72]).

In medium and LPP systems, the most important inactivation mechanism is DNA destruction by UV irradiation, followed by erosion of microbial material by intrinsic photodesorption and etching with reactive species, possibly enhanced by UV photons [72,94]. Hence, UV radiation makes a significant contribution to sterilization in LPP. The biological action behind this is well known: UV radiation induces the formation of thymine dimers in DNA, inhibiting its ability to replicate [93]. A decisive weakness of this type of plasma sterilization is that the penetration of UV photons is limited and the inactivation efficiency thus depends on the thickness of the material (microorganisms): UV photons must reach the DNA in the cells in order to effectively destroy it [94]. The primary role of UV photons in microbial inactivation in LPP was confirmed by other authors [95,96,97,98,99,100]. However, different research groups have observed large differences in sterilization kinetics (shapes of survival curves) in LPP sterilization techniques, which suggests that there are different mechanisms responsible for sterilization, probably due to different conditions of plasma generation [92]. Also, when considering the antimicrobial efficacy of the LPP system, the vast majority of experiments was performed on bacterial cells and spores. However it has been proposed that fungi could be more resistant to external stress (such as UV) due to a protective layer of pigment melanin in their cell walls [101].

Although LPP have brought a significant improvement over traditional sterilization methods, they still have some shortcomings, such as the need for vacuum, long processing times, high costs, and the need for batch processing [92]. For this reason, studies in recent years have focused mainly on research of atmospheric pressure (AP) plasmas, where disadvantages of LPP are overcome and work at much higher pressures is enabled.

#### 3.3.2. Effects of Atmospheric-Pressure Plasmas (APP)

In contrast to plasma systems with reduced pressure, the UV photons in atmospheric or high-pressure plasma systems have not been attributed a significant role in the sterilization process [5,92]. This is because the gas mixtures used in APP systems do not emit a sufficient dose of UV radiation at the germicidal wavelengths, while shorter generated wavelengths do not penetrate depth enough to cause lethal damage to microorganisms [93]. Compared to LPP, sterilization with APP is characterized by more sophisticated mechanisms and kinetics, namely the gas-phase collisions, which take place at higher pressure and produce a wider range of active species involved in the sterilization process [102]. The energies of electrons are much higher than those of heavy particles (ions, neutrals), and these electrons collide with atoms and molecules, resulting in an increased degree of dissociation, excitation, and ionization of molecules [34].

In high-pressure plasma, the resulting reactive species play an important role in microbial inactivation. It is assumed that different reactive oxygen species (ROS) and reactive nitrogen species (RNS), such as atomic oxygen (O), metastable oxygen (O_2_*), superoxide (O_2_^−^), ozone (O_3_), hydroxyl radical (OH^−^), hydrogen peroxide (H_2_O_2_), nitride oxide (NO), and nitride dioxide (NO_2_) play a key role in the sterilization process [34,74,78,93,102,103,104,105,106,107].

**Figure 3:**
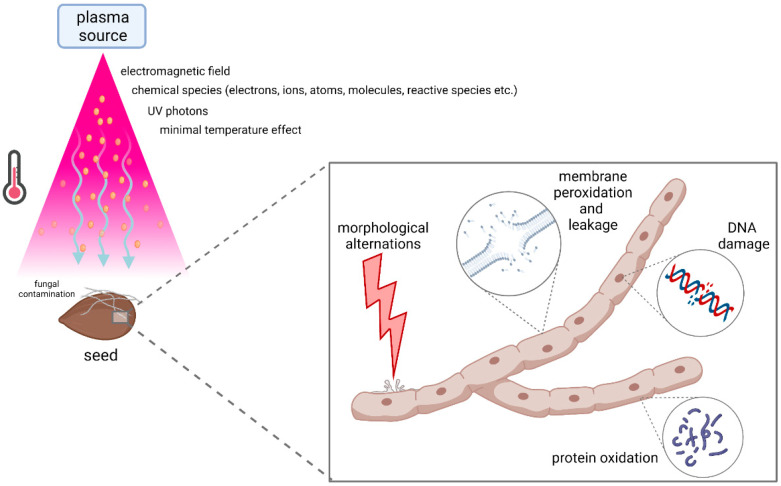
Main agents and mechanisms governing fungal decontamination in cold plasma sterilization process. This image was created with BioRender.com.

Oxygen-containing plasma mixtures were shown to have a particularly increased germicidal effect [108,109,110]. One of the first and most comprehensive studies investigated bacterial spore (from genus *Bacillus*) inactivation by AP DBD in two different gas mixtures: pure helium and a mixture of 97% helium and 3% oxygen. After 10 min of plasma treatment, the survival rate of the spores treated with pure helium was about 70%, while a much higher killing rate was observed in the helium/oxygen mixture, with less than 10% of the spores surviving. This large difference in inactivation efficacy can thus be attributed to the presence of a variety of reactive oxygen species that are generated in the plasma (such as O and O_3_) when oxygen is added to the gas mixture [111]. A major role in the inactivation process of *Aspergillus oryzae* and *Penicillium digitatum* was attributed to atomic oxygen, resulting in morphological changes in fungal hyphae [112]. Morphological damages of fungal structures were also reported in a study where *Aspergillus flavus* cells were treated with RF plasma jet, resulting in cell leakage and lower viability [113]. Similar results were obtained by AP DBD CP treatment of *Aschochyta pinodella* and *Fusarium culmorum*, where damages in cell walls and cell membranes resulted in cell cytoplasm leakage [114]. The main mechanisms behind CP-induced fungal inactivation process are shown in Figure 3.

Reactive species interact with living cells and their macromolecules and influence their structural and biochemical properties. The inactivation of microorganisms and their spores under APP has been attributed mainly to ROS and RNS [78,105], as they can directly impact the cell walls and outer membranes of microorganisms [34,74]. They could break the structurally important chemical bonds of the peptidoglycan layer, leading to destruction of the bacterial cell wall [107]. Furthermore, charged particles can also rupture the outer bacterial membrane [115]. Reactive species cause alterations in cellular macromolecules, with membrane lipids being the most vulnerable group as they are located near the cell surface and are thus most sensitive to ROS [116]. The CP-generated ROS were also attributed a major role in physiological changes, especially oxidation of intracellular organelles, particularly lipids and cellular proteins [117,118,119].

Montie et al., 2000 [116] examined the effect of APP on microbial inactivation and structural changes in Gram-negative (*E. coli*) and Gram-positive bacteria (*S. aureus*). The *E. coli* cells were rapidly and severely damaged, exhibiting significant macromolecular leakage caused by a rupture of the outer membrane (probably due to peroxidation of fatty acids). In contrast, *S. aureus* cells showed no morphological changes and cell leakage was delayed. This was probably due to the thick polysaccharide cell wall layer characteristic of Gram-positive bacteria, protecting them from ROS attack, but still allowing ROS diffusion and attack on the cytoplasmic membrane, causing cell leakage. In both cases, macromolecules are released, which leads to death of bacterial cells, with *S. aureus* still retaining its spherical shape due to the thick peptidoglycan cell wall layer. Similar results were later confirmed by Laroussi et al., 2003 [120], who investigated the effect of APP on Gram-negative (*E. coli*) and Gram-positive bacteria (*B. subtilis*). These findings show that spores and yeasts are the most resistant structures to plasma treatment due to a very thick layer of polysaccharides in the cell wall [116]. Morphological changes resulting in cell wall and membrane alterations that lead to increased permeability and cell leakage were also reported for fungal cells and spores of *Aspergillus* sp. [83,84] and spores of *Neurospora crassa*, [117]. Three main killing mechanisms of APP were proposed: (1) Lipid peroxidation, as a result of hydroxyl radicals attacking unsaturated fatty acids. (2) Protein oxidation, due to amino acid oxidation. (3) DNA oxidation, caused by interactions with oxygen radicals, leading to the formation of base adducts (Adapted from [116]). Destruction of DNA of fungal spores (*Cordyceps bassiana*) treated with APP was also confirmed [121].

Examination of different gas mixtures led to a conclusion that the best bactericidal effects are achieved in moistened oxygen and air [110]. In the mixture, humidity leads to the formation of OH radicals that attack the external structures of bacterial cells; oxygen produces ozone (O_3_) that interferes with cellular respiration; and air contains nitrogen that leads to the production of NO and NO*x*, which contribute to the lethality of the process, leading to more efficient microbial inactivation. AP air-gas mixture plasma is thus a very efficient sterilizing agent. The role of RNS in fungal inactivation is less known and still remains to be examined.

APP systems are particularly useful and have a key advantage over LP plasmas, as they do not require large vacuum devices and therefore offer the possibility of in-line batch processing of seeds [64]. In addition, they have many potential advantages over conventional sterilization methods because they are non-toxic, have low operating costs and a short treatment time at low temperatures, do not use water in the process, and can be used for a wide range of goods [122,123,124].

## 4. Conclusions & Future Research

CP is a prominent method for seed treatment, especially the AP CP systems, as they do not require large vacuum chambers and provide in-line batch processing of grain. In addition, the use of air as the feed gas for plasma generation provides the most effective results in terms of seed surface changes, such as microbial decontamination and increased wettability, and thus, increased water absorption due to changes in contact angle. The introduction of novel, environmentally friendly alternatives for seed treatment could also reduce undesirable residues of xenobiotics such as organic fungicides, which are now widely used in crop protection both in plants and in the environment. The reviewed papers show that CP could be used as an alternative method for fungal decontamination of seeds. CP is also relatively safe for work, both for the operator and the treated subject, if the treating parameters are optimized. Likewise, it requires relatively low energy input and can therefore be an economically and environmentally sustainable method for decontamination. However, it is hard to compare various studies as plasma types and parameters used are quite diverse. There is also a lack of systematic studies on the effects of different CP treatments on various types of seeds and under different environmental conditions. It is already clear from the studies carried out to date, that the effectiveness of different CP treatments on fungal decontamination, seed germination, and plant growth depends on the type of seed (plant species), storage conditions, and also on environmental factors. In this perspective, there is a lack of field studies involving plasma-treated seeds.

Up to this point, there is still not enough data to decide which CP treatment method is effective in improving seed germination. The same applies to the effectiveness of CP treatment on microbial inactivation and thus on seed decontamination, as the plasma setups and parameters used in the different studies are quite varied. Since it was observed in most studies that complete inactivation of microorganisms on the seed surface generally requires longer plasma exposure times, which can cause damage and lead to inhibition of germination, it appears that plasma treatment may be useful for decontaminating seeds intended for storage or feeding. In the case of seeds intended for planting and cultivation of crops, shorter exposure times should be used to achieve beneficial effects on germination and growth parameters. In addition, it is obvious that not only each type of seed, but probably each pathogen species, requires different plasma exposure times for complete inhibition, as sensitivity to plasma treatment seems to be species specific. There is also a lack of data on the effect of CP treatment on seed endophytic fungi (both beneficial and pathogenic).

Future research should therefore focus on the unification of optimization of CP treatment parameters for economically important crops and their pathogens, in particular various types of filamentous fungi, known to contaminate seeds during storage and being harmful to both seeds and humans, in order to ensure global food security. The use of plasma techniques could also be beneficial in applications with horticultural as well as rare and endangered plant species, and it should only be a few years until large scale CP will be used for seed treatment.

## Figures and Tables

**Table 1:** An overview of experiments studying the effects of cold plasma on fungal decontamination in various plant species. AI: Artificially infected. NO: naturally occurring. CP: cold plasma. LP: low-pressure. AP: atmospheric-pressure. SMD: Surface micro-discharge. FB: fluidized bed. DCSBD: Diffuse Coplanar Surface Barrier Discharge. RF CC: radio-frequency capacitively coupled. (S)DBD: (Surface) Dielectric Barrier Discharge. CDPJ: Corona Discharge Plasma Jet. P: power. F: frequency. V: voltage. SPD: surface power density. PVD: power volume density. TT: treatment time. NT: not tested.

Fungal Species	Seed Type	Plasma Source and Properties	Gas Type and Exposure Time	Key Findings	% of Germination	Ref.
AI *Aspergillus* spp. and *Penicillum* spp.	Tomato (*Lycopersicon esculentum*), wheat (*Triticum durum*), bean (*Phaseolus vulgaris*), chick pea (*Cicer arietinum*), soybean (*Glycine max*) barley (*Hordeum vulgare*), oat (*Avena sativa*), rye (*Secale cereal*), lentil (*Lens culinaris*), and corn (*Zea mays*)	LP CP (500 mTorr); P: 300 W; F: 1 kHz; V: 20 kV	Air gas and SF_6_; 5–20 min	Significant reduction to below 1% of initial fungal load	No significant effect on wheat and bean seeds	[6]
AI *Aspergillus parasiticus*	Hazelnuts (*Corylus avellane*), peanuts (*Arachis hypogaea*) and pistachio nuts (*Pistacia vera*)	LP CP (500 mTorr); P: 300 W; F: 1 kHz; V: 20 kV	Air gas and SF_6_; 1–20 min	Air plasma 1-log reduction of initial load, SF_6_ plasma more effective with app. 5-log decrease (after 5 min TT)	NT	[70]
NO microbiota	Chickpea (*Cicer arietinum*)	AP SMD CP; SPD: 10 mW/cm^2^; V: 5–17 kV	Ambient air; 0.5–5 min	Significant reduction of 1–2 log of microbial contamination	Increased up to 3 min TT	[4]
AI *Aspergillus flavus* and *A. parasiticus*	Hazelnuts (*Corylus avellane*)	AP FB CP; P: 460–655 W; F: 25 kHz; V: 5–10 kV	Air gas and N_2_; 1–5 min	Significant reduction of app. 4-log (CFU/g) after 5 min of air gas plasma TT	NT	[83,84]
AI *Fusarium circinatum* (pine pest)	Pine (*Pinus radiata*)	AP DCSBD CP; F: 14 kHz; V: 10 kV	Air gas; 5–300 s	Reduction of seedborne pathogens (14–100%)	No significant effect	[85]
NO microbiota and AI *Fusarium nivale, F. culmorum, Trichothecium roseum, A. flavus, A. clavatus*	Wheat (*Triticum aestivum*)	AP DCSBD CP; PVD: 100 W/cm^3^; P: 400 W	Ambient air; 10–600 s for NO microflora; 1–300 s for AI fungi-infected seeds	Increased inhibition of microflora with increased treatment time; Total devitalization of NO filamentous fungi after 120 s TT	Increased up to 40 s TT, then decreased	[18]
NO microbiota and AI *A. flavus, Alternaria alternata, F. culmorum*	Maize (*Zea mays*)	AP DCSBD CP; PVD: 80 W/cm^3^; P: 400 W	Ambient air; 60–300 s	Total devitalization of NO bacteria after 60 s and NO fungi 180 s TT	No significant effect up to 120 s TT, then decreased	[51]
NO fungi (*Fusarium, Alternaria, Stemphylium*)	Wheat (*Triticum aestivum*), spring barley (*Hordeum vulgare*), blue lupine (*Lupinus angustifolius*), soy (*Glycine soja*), and field pea (*Pisum arvense*)	AP RF CC CP; PVD: 0.6 W/cm^3^; F: 5.28 MHz	Air gas; 2–20 min	Reduction of fungal infection; the most effective TT at 10 and 15 min	Little enhancement in blue lupine and field pea	[86]
NO fungal microbiota	Wheat (*Triticum aestivum*)	AP CP; F: 100 Hz–83 kHz; V: 8 kV	Air gas; 3–30 s	Reduction of fungal colonies on wheat grains at the optimum 10 s TT	No significant effect	[87]
NO microbiota	Sweet basil (*Ocimum basilicum*)	AP SDBD CP; SPD: 80 mW/cm^2^; F: 5 kHz	Humid air; 10–600 s	Significant decrease in microbial load (up to 50% in 300 s TT)	No significant effect	[88]
NO microbiota (molds and yeasts)	Rapeseed (*Brasica napus*)	AP CDPJ CP; F: 58 kHz V: 20 kV, 58 kHz	Air gas; 0.5–3 min	Reduction by 2-log units compared to initial count	Positive effect up to 1 min TT	[61]
NO bacteria and fungi	Ginseng (*Panax ginseng*)	AP DBD; F: 60 Hz; V: 120 V	Ar and Ar/O_2_ mixture (80:20); 10 min each day, 3 days in a row	Ar/O_2_ plasma mixture had better bactericidal and fungicidal effect	Positive effect in both mixtures	[89]
NO fungi (molds and yeasts)	Common buckwheat (*Fagopyrum esculentum*) and Tartary buckwheat (*F. tataricum*)	LP RF CP (50 Pa); P: 1400 W; F: 27.12 MHz	Pure O_2_ plasma; 30–120 s	Reduction of seedborne fungi to below 50% of control after 120 s	No significant effect up to 45 s TT, then decreased	[33]
